# Pyocyanin-dependent electrochemical inhibition of *Pseudomonas aeruginosa* biofilms is synergistic with antibiotic treatment

**DOI:** 10.1128/mbio.00702-23

**Published:** 2023-06-14

**Authors:** Fernanda Jiménez Otero, Dianne K. Newman, Leonard M. Tender

**Affiliations:** 1 College of Science, George Mason University, Fairfax, Virginia, USA; 2 Biology and Biological Engineering and Geological and Planetary Sciences, Caltech, Pasadena, California, USA; 3 Center for Bio/Molecular Science and Engineering, US Naval Research Laboratory, Washington, DC, USA; Universite de Geneve, Geneva, Switzerland

**Keywords:** *Pseudomonas aeruginosa*, biofilms, pyocyanin, electrochemistry, antibiotics

## Abstract

*Pseudomonas aeruginosa* biofilms are common in chronic wound infections and recalcitrant to treatment. Survival of cells within oxygen-limited regions in these biofilms is enabled by extracellular electron transfer (EET), whereby small redox active molecules act as electron shuttles to access distal oxidants. Here, we report that electrochemically controlling the redox state of these electron shuttles, specifically pyocyanin (PYO), can impact cell survival within anaerobic *P. aeruginosa* biofilms and can act synergistically with antibiotic treatment. Prior results demonstrated that under anoxic conditions, an electrode poised at sufficiently oxidizing potential (+100 mV vs Ag/AgCl) promotes EET within *P. aeruginosa* biofilms by re-oxidizing PYO for reuse by the cells. Here, when a reducing potential (−400 mV vs Ag/AgCl) was used to disrupt PYO redox cycling by maintaining PYO in the reduced state, we observed a 100-fold decrease in colony forming units within these biofilms compared with those exposed to electrodes poised at +100 mV vs Ag/AgCl. Phenazine-deficient Δphz* biofilms were unaffected by the potential applied to the electrode but were re-sensitized by adding PYO. The effect at −400 mV was exacerbated when biofilms were treated with sub-MICs of a range of antibiotics. Most notably, addition of the aminoglycoside gentamicin in a reductive environment almost completely eradicated wild-type biofilms but had no effect on the survival of Δphz* biofilms in the absence of phenazines. These data suggest that antibiotic treatment combined with the electrochemical disruption of PYO redox cycling, either through the toxicity of accumulated reduced PYO or the disruption of EET, or both, can lead to extensive killing.

**IMPORTANCE:**

Biofilms provide a protective environment but also present challenges to the cells living within them, such as overcoming nutrient and oxygen diffusion limitations. *Pseudomonas aeruginosa* overcomes oxygen limitation by secreting soluble redox active phenazines, which act as electron shuttles to distal oxygen. Here, we show that electrochemically blocking the re-oxidation of one of these electron shuttles, pyocyanin, decreases cell survival within biofilms and acts synergistically with gentamicin to kill cells. Our results highlight the importance of the role that the redox cycling of electron shuttles fulfills within *P. aeruginosa* biofilms.

## OBSERVATION

Biofilms provide bacterial cells with a protective environment where persistence and antibiotic tolerance arise, making them a leading contributor to chronic infections (1). Extracellular electron transfer (EET) pathways have been recurrently found among biofilm-forming opportunistic pathogens (2 - 4). Such pathways are often dependent on the redox cycling of either self-made or borrowed small molecules that serve as electron shuttles between cells in the biofilm and extracellular terminal electron acceptors (5). Specifically, in the biofilms formed by *Pseudomonas aeruginosa* PA14 (6), oxygen limitation within anoxic regions is overcome through the use of phenazines as electron shuttles to reduce distal oxygen (7, 8). Of the different phenazines produced by *P. aeruginosa* PA14, pyocyanin (PYO) is present at high abundance and facilitates EET via its association with extracellular DNA in the biofilm matrix (9).

Electrochemical control over biofilms has been explored in several ways in the past. Applying a weak current to biofilms formed on electrodes by persister *P. aeruginosa* PAO1 cells decreases cell survival, but the mechanism underpinning this observation is not understood (10). Electric bandages poised at −600 mV vs Ag/AgCl to produce H_2_O_2_ also decrease cell survival within multi-species biofilms (11). However, H_2_O_2_ has been known to prolong the wound healing process and is cytotoxic at high concentrations, which may be counteractive to its antimicrobial effects (12). Providing a poised electrode as an alternative electron acceptor in the proximity of agar-grown *P. aeruginosa* PA14 colonies delays wrinkling colony morphology associated with the development of oxygen-limited regions by alleviating oxidant limitation (13). Additionally, biochemically altering PYO through demethylation has also been effective in decreasing *P. aeruginosa* PA14 cell survival and is synergistic with antibiotic treatment (14).

**Fig 1:**
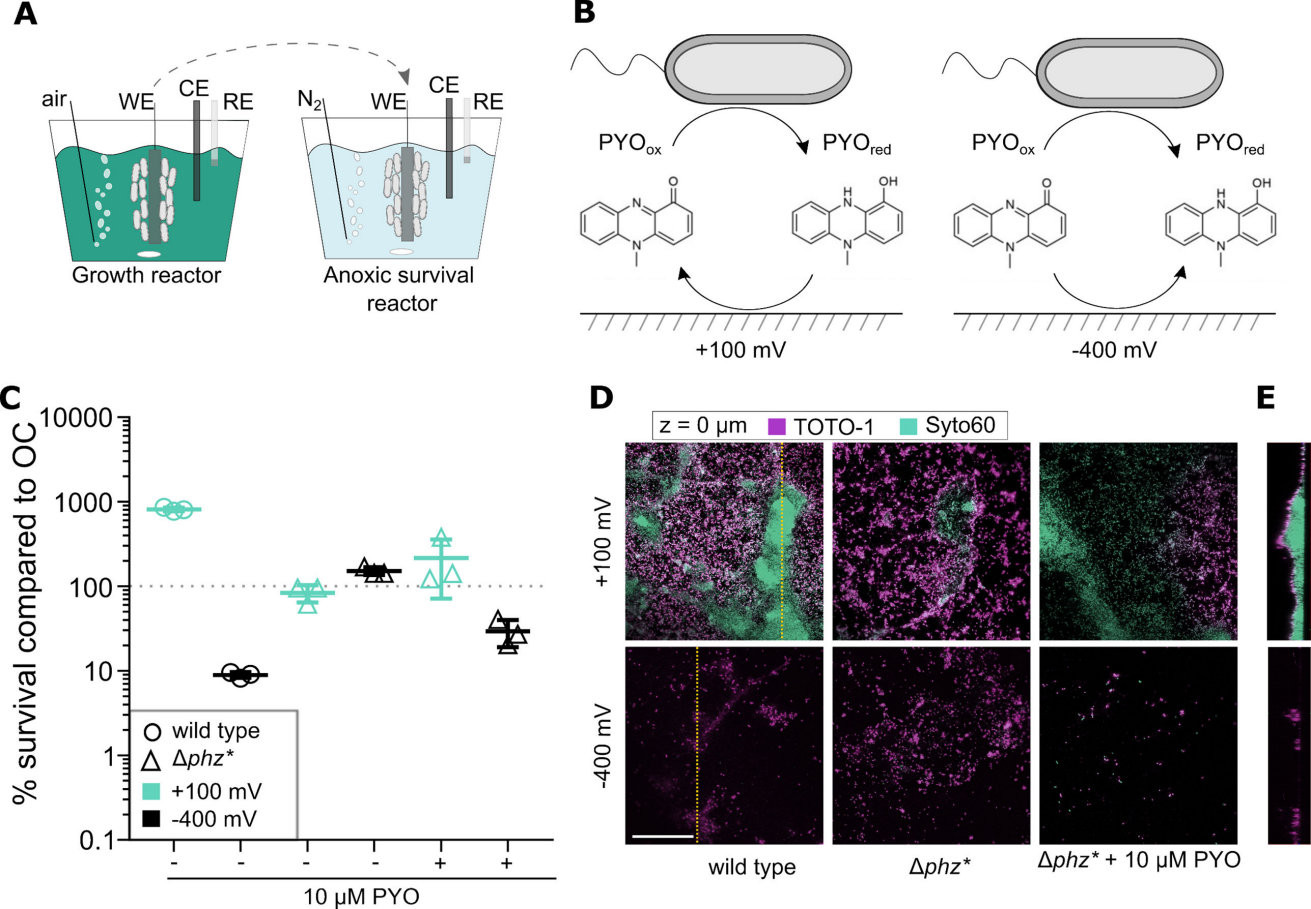
EET impacts cell survival and biofilm morphology. (**A**) Schematic representation of experimental setup. Working electrodes served as biofilm attachment surfaces that were at open circuit (neither oxidizes nor reduces PYO) or poised at the PYO-oxidizing potential of +100 mV or at the PYO-reducing potential of −400 mV vs Ag/AgCl. Growth reactors were incubated under oxic conditions for 5 d with fresh medium exchanged every 24 h. The working electrodes were then transferred to anoxic survival reactors that were flushed with N_2_ gas and incubated for 72 h before harvesting and processing biofilm. WE, working electrode; CE, counter electrode; RE, reference electrode. (**B**) Schematic representation of redox cycling of PYO between cells that reduce PYO_ox_ to PYO_red_ and the electrode under PYO-oxidative (+100 mV) and PYO-reductive (−400 mV) conditions; under PYO-oxidative conditions, PYO_red_ is oxidized to PYO_ox_, allowing redox cycling to proceed; under PYO-reductive conditions, PYO_ox_ is reduced to PYO_red_, thereby breaking the cycle. (**C**) CFUs after 72 h under anoxic conditions (*n* =3) normalized to parallel OC negative control samples (see raw data in Fig. 2) for wild type, Δphz*, and Δphz* + 10 µM PYO biofilms. Error bars represent standard error. (**D**) Fluorescence microscopy images of biofilm interface with electrode surface using TOTO-1 (cell-impermeable, eDNA) and Syto60 (cell-permeable, all DNA) from samples representative of three fields of view from triplicate cultures. Bar = 50 μm. (**E**) Vertical slice of complete z-stack along yellow dotted lines shown in wild-type panels in (D).

Here, we report that electrochemically disrupting redox cycling under anoxic conditions can inhibit cell survival. *P. aeruginosa* PA14 biofilms were grown for 5 days in actively aerated three-electrode electrochemical reactors using an indium tin oxide (ITO)-covered glass slide as both a biofilm attachment surface and transparent working electrode (Fig. 1A). In contrast to previous studies (15), under our conditions, the main phenazine detected was PYO (Fig. S1 and Detailed Experimental Procedures). Electrode-attached biofilms were then transferred to anoxic reactors for 72 h (Fig. 1A), after which the biofilms were harvested for colony forming unit (CFU) counts (Fig. 1C) and biofilm imaging (Fig. 1D). During both growth and after transfer to the anoxic reactors, the ITO working electrodes were poised at either the PYO-oxidative potential of +100 mV vs Ag/AgCl, or the PYO-reductive potential of −400 mV vs Ag/AgCl, which is not low enough to produce H_2_O_2_ in the presence of oxygen (16). Under these conditions, for anoxic reactors in which the electrode was poised at +100 mV vs Ag/AgCl, PYO redox cycling can occur, but not in reactors in which the electrode was poised at −400 mV vs Ag/AgCl (Fig. 1B), except possibly at a low level if trace oxygen was present. Additional “untreated” control biofilms were set to open circuit (OC) in which no potential was applied to the electrode.

**Fig 2:**
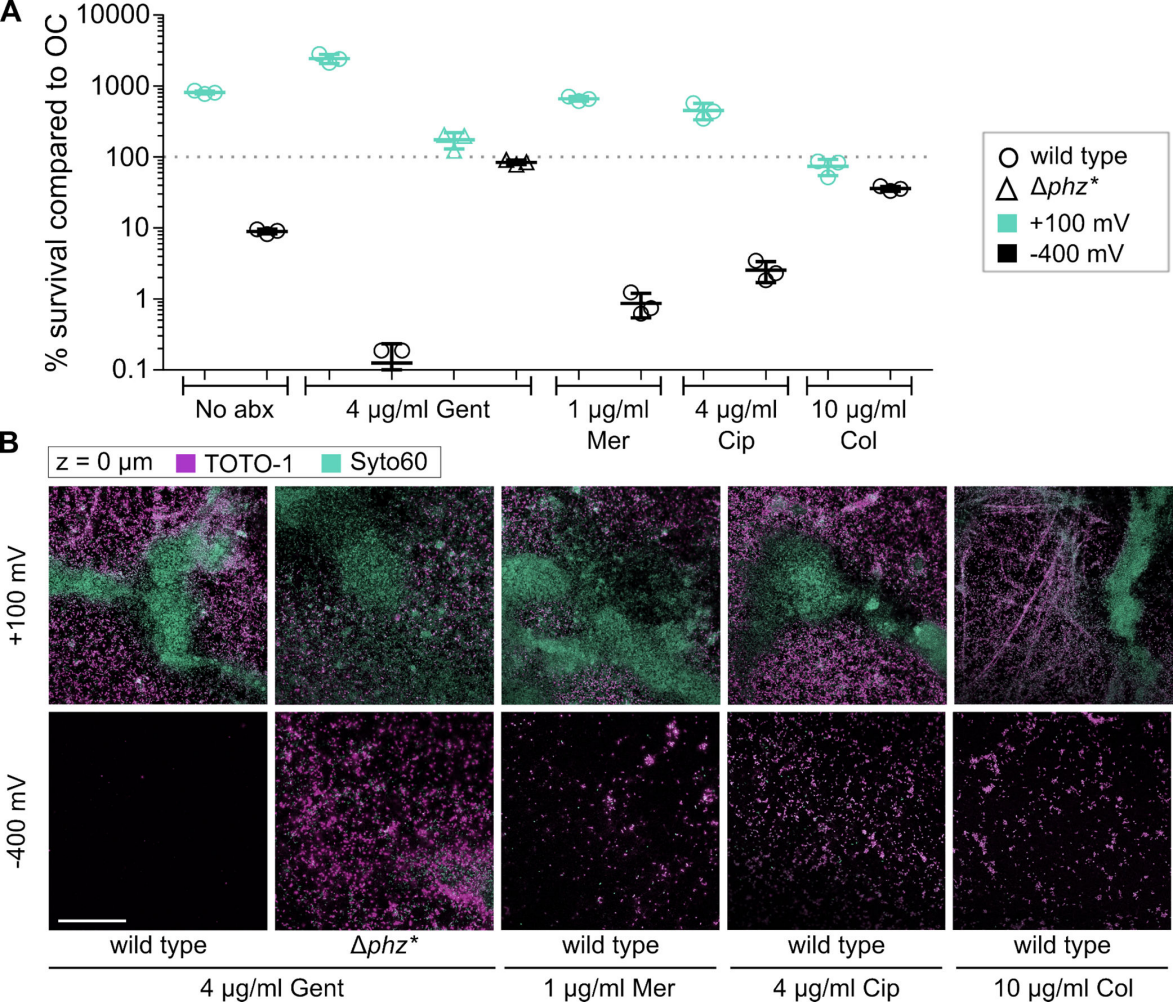
Reduced PYO acts synergistically with antibiotic treatment. (**A**) CFUs after 72 h under anoxic conditions normalized to parallel OC samples (see raw data in Fig. S4) in the presence of either 4 µg/mL gentamicin (Gent), 1 µg/mL meropenem (Mer), 4 µg/mL ciprofloxacin (Cir), or 10 µg/mL colistin (Col), *n* = 3. Error bars represent standard error. Data from samples not treated with antibiotics (No abx) from Fig. 1 plotted again for ease of comparison. (**B**) Fluorescence microscopy images of biofilm interface with electrode surface using TOTO-1 (cell-impermeable, eDNA) and Syto60 (cell-permeable, all DNA) from representative samples shown in (A). Bar = 50 μm.

### Electrochemically blocking pyocyanin re-oxidation during anoxic conditions decreases cell survival

Compared with OC control conditions, anoxic PYO-oxidative conditions enhanced cell survival by 10-fold while PYO-reductive conditions decreased wild-type (WT) cell survival by 10-fold (CFUs/cm^2^ ± standard error [SE], *n* = 3, for OC = [2.35 ± 0.11] × 10^4^, +100 mV = [1.91 ± 0.06] × 10^5^, and −400 mV = [2.10 ± 0.09] × 10^3^, Fig. 1C and Fig. S2]. This electrode potential-dependent effect on survivability was also observed for biofilms grown on glass surfaces placed ~3 cm from the working electrode (Fig. S3), suggesting that redox cycling enabled by an electrode at a distance can support cell survival, as expected from previous studies in planktonic culture (7). CFUs of phenazine-deficient Δphz* biofilms grown without PYO were not affected by the potential applied to the electrodes but were sensitized under PYO-reductive conditions by the addition of 10 µM PYO, indicating that cell survival in this context is PYO mediated (Fig. 1 and Fig. S1).

Biofilm morphology was qualitatively consistent with results from CFU counts, with biofilms treated under PYO-oxidative conditions showing full electrode surface coverage and secondary structures up to 100 µm thick; large microcolonies stained brightly with SYTO 60 in the core yet took up TOTO-1 in the periphery (Fig. 1D and E). As these dyes provide a measure of cell permeability as well as staining extracellular DNA (TOTO-1), consistent with previous studies (17), we interpret these results to indicate that cells in the interior were intact, whereas those on the periphery had compromised membranes. In comparison, biofilms treated under PYO-reductive conditions were made up of single-cell layers with no secondary structures and a greater proportion of membrane-permeable cells (Fig. 1D and E). This pattern held true for all samples. Addition of PYO to Δphz* biofilms did not fully recapitulate the WT morphology possibly due to a lower amount of extracellular DNA in the biofilm matrix; eDNA release has been shown to be stimulated by PYO production (18), and eDNA is also necessary for PYO retention (9).

### Reduced PYO acts synergistically with antibiotics to kill cells

Sub-MICs of gentamicin, meropenem, ciprofloxacin, and colistin were added to anoxic survival reactors. Most notably, the addition of 4 µg/mL of gentamicin to PYO-reductive conditions almost fully eradicated WT biofilms (CFU/cm^2^ ± SE, *n* = 3, for OC = [1.71 ± 0.59] × 10^4^, –400 mV = 21.4 ± 10.7) but did not affect Δphz* biofilms. As PYO has been shown to confer tolerance to aminoglycosides (19), our data suggest that oxidized PYO confers tolerance to aminoglycosides, which is also disabled by PYO that is biochemically altered (14). Alternatively, or in addition, our data are consistent with previous reports indicating reduced phenazines can be toxic in the presence of a sufficient concentration of iron in the medium to trigger the formation of phenazine radical species (20); assuming such reactions were also at play under our conditions, this mode of toxicity may have contributed to causing cell death when combined with aminoglycosides. Treatment with 1 µg/mL meropenem (a β-lactam) or 4 µg/mL ciprofloxacin (a fluoroquinolone) under PYO-reductive conditions also decreased cell survival compared with OC by ~100× (CFU/cm^2^ ± SE, *n* = 3, for meropenem OC = [7.72 ± 0.95] × 10^4^, –400 mV = [6.73 ± 1.47] × 10^2^; for ciprofloxacin OC = [1.39 ± 0.11] × 10^4^, –400 mV = [3.53 ± 0.66] × 10^2^) but cell clusters sparsely covering the electrode surface were still present (Fig. 2B). Consistent with our results, previous reports have shown that PYO confers tolerance to ciprofloxacin and the β-lactam carbenicillin, with Δphz* cells in either colony biofilms or liquid cultures being more susceptible to ciprofloxacin and carbenicillin than wild type (19, 21). Future studies will investigate how antibiotic treatment in the absence of phenazines compares with the conditions presented here, where PYO is present but in a reduced state. Treatment with 10 µg/mL colistin did not show an additive effect with PYO-reductive conditions, yet PYO-oxidative conditions in the presence of colistin showed a 10× decrease in CFUs compared with nontreated biofilms (CFU/cm^2^ ± SE, *n* = 3, +100 mV + 10 µg/mL colistin = [1.74 ± 0.15] × 10^4^, +100 mV no antibiotic = [1.91 ± 0.06] × 10^5^), a finding consistent with a previous study showing that colistin synergizes with phenazines to kill cells in colony biofilms under oxic conditions (19). Based on our results under anoxic conditions with cell-permeability dyes, we hypothesize PYO-oxidative conditions are most likely to mimic those within colony biofilms since a larger proportion of metabolically active cells arises when oxidized PYO is available (Fig. 2B), and this resembles what we would expect for cells within colony biofilms grown under oxic conditions (22).

To characterize possible toxic effects of reduced PYO on cell survival, biofilms were harvested pretransfer under oxic conditions and after 30 min, 6, 36, and 72 h after transfer to anoxic conditions. Plating was done in parallel on both oxic and anoxic media to rule out the effects of experimental setup on cell death. We observed no significant difference between CFUs of biofilms pretransfer (oxic) at −400 mV vs Ag/AgCl and +100 mV vs Ag/AgCl. After 30 min from transferring to anoxic reactors, CFUs from biofilms grown under PYO-reductive conditions decreased 100-fold compared with original aerobic biofilms, while PYO-oxidative conditions only caused a slight decrease in CFUs (Fig. S5). While such rapid killing is consistent with PYO toxicity, under anoxic PYO-reductive conditions, EET is also disrupted in biofilms previously grown under oxic conditions. We, therefore, used mid-log aerobic cell cultures to inoculate anoxic medium containing a biochemical O_2_ scavenging system for which EET is not possible in the absence of both an electrode or O_2_. Increasing concentrations of reduced PYO led to a two- to threefold decrease in CFUs (Fig. S6). However, increasing concentrations of reduced PYO did not correlate with a decrease in CFUs and did not achieve the 10-fold decrease seen in biofilm experiments between OC and PYO-reductive conditions. The discrepancy between liquid culture and biofilm experiments may be due to (i) an antitoxicity pathway present in fresh liquid cultures but unexpressed in cells within week-old biofilms or (ii) the presence of a working electrode constantly driving the PYO pool toward a fully reduced state or creating secondary toxic products at the biofilm attachment surface. Alternatively, it may point to EET disruption as being more critical for biofilm cell death than generation of reduced PYO *per se*.

Taken together, our results highlight the importance of redox cycling for *P. aeruginosa* survival within oxygen-limited biofilms and demonstrate that electrochemical manipulation, in tandem with antibiotic treatment, can be applied to better control biofilms of opportunistic pathogens. As redox cycling both promotes EET and decreases the amount of reduced PYO in a *P. aeruginosa* biofilm, it is not possible to determine from the observations reported here which one has the biggest effect on biofilm cell survivability; this unknown will be addressed in future studies. However, this work provides context for the mechanism behind previous observations of cell death in the presence of a weak electric current (10) and provides conditions under which existing electrical bandage technology (11) may be modified to become more host compatible. Finally, several novel research queries are also posed by the data presented here, such as characterizing the mechanism of toxicity of reduced PYO and how it synergizes or antagonizes the effect of particular antibiotics as well as how electrochemically blocking electron shuttle cycling may impact other organisms found in polymicrobial infections in close proximity to *P. aeruginosa*.

## SUPPLEMENTAL MATERIAL

Fig. S1 mbio.00702-23-s0001.docx Square wave voltammograms.

Fig. S2 mbio.00702-23-s0002.docx Un-normalized data from Fig. 1.

Fig. S3 mbio.00702-23-s0003.docx Cell survival within biofilms.

Fig. S4 mbio.00702-23-s0004.docx Un-normalized data from Fig. 2.

Fig. S5 mbio.00702-23-s0005.docx Cell survival over time.

Fig. S6 mbio.00702-23-s0006.docx Planktonic culture PYO toxicity.

TABLE S1 mbio.00702-23-s0007.docx Strains used.

Supplemental Material mbio.00702-23-s0008.docx Detailed experimental procedures.

ASM does not own the copyrights to Supplemental Material that may be linked to, or accessed through, an article. The authors have granted ASM a non-exclusive, world-wide license to publish the Supplemental Material files. Please contact the corresponding author directly for reuse.
