# Supplementary material for: Pyocyanin-dependent electrochemical inhibition of Pseudomonas aeruginosa biofilms is synergistic with antibiotic treatment
Source: mBio. 2023 Jun 14;14(4):e00702-23. doi: 10.1128/mbio.00702-23 (PMC10470778; doi:10.1128/mbio.00702-23)
Supplement: Fig. S1 — Square wave voltammograms. [file mbio.00702-23-s0001.docx]

**Supplemental Figure S1**

**Figure S1**. Electrochemical signal of wild type, Δ*phz**, and Δ*phz**+ 10µM PYO showing PYO (E_1/2_ = -250 mV vs Ag/AgCl) is the only redox active signal observed under these conditions and is retained by wild type biofilms. Square wave voltammograms acquired immediately after transfer to anoxic reactors.
