# Supplementary material for: Pyocyanin-dependent electrochemical inhibition of Pseudomonas aeruginosa biofilms is synergistic with antibiotic treatment
Source: mBio. 2023 Jun 14;14(4):e00702-23. doi: 10.1128/mbio.00702-23 (PMC10470778; doi:10.1128/mbio.00702-23)
Supplement: Fig. S2 — Un-normalized data from Fig. 1. [file mbio.00702-23-s0002.docx]

**Supplemental Figure S2**


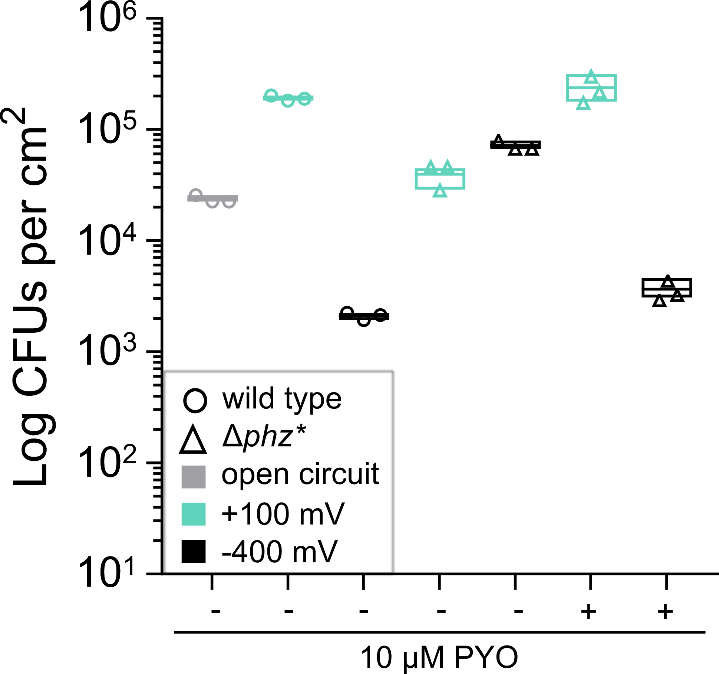


**Figure S2**. Un-normalized data from Fig. 1. CFUs after 72 hours under anoxic conditions, n=3. Box plots represent standard error.
