# Supplementary material for: Pyocyanin-dependent electrochemical inhibition of Pseudomonas aeruginosa biofilms is synergistic with antibiotic treatment
Source: mBio. 2023 Jun 14;14(4):e00702-23. doi: 10.1128/mbio.00702-23 (PMC10470778; doi:10.1128/mbio.00702-23)
Supplement: Fig. S3 — Cell survival within biofilms. [file mbio.00702-23-s0003.docx]

**Supplemental Figure S3**

**Figure S3**. Cell survival within biofilms exposed to PYO-reductive or PYO-oxidative conditions at a distance of ~3cm. CFUs from biofilms grown on electrodes poised at OC shown in gray, poised at +100 mV (line) and unpoised (dashed) within the same reactor shown in teal, and poised at -400 mV (line) and unpoised (dashed) within same reactor shown in black.
