# Supplementary material for: Pyocyanin-dependent electrochemical inhibition of Pseudomonas aeruginosa biofilms is synergistic with antibiotic treatment
Source: mBio. 2023 Jun 14;14(4):e00702-23. doi: 10.1128/mbio.00702-23 (PMC10470778; doi:10.1128/mbio.00702-23)
Supplement: Fig. S4 — Un-normalized data from Fig. 2. [file mbio.00702-23-s0004.docx]

**Supplemental Figure S4**


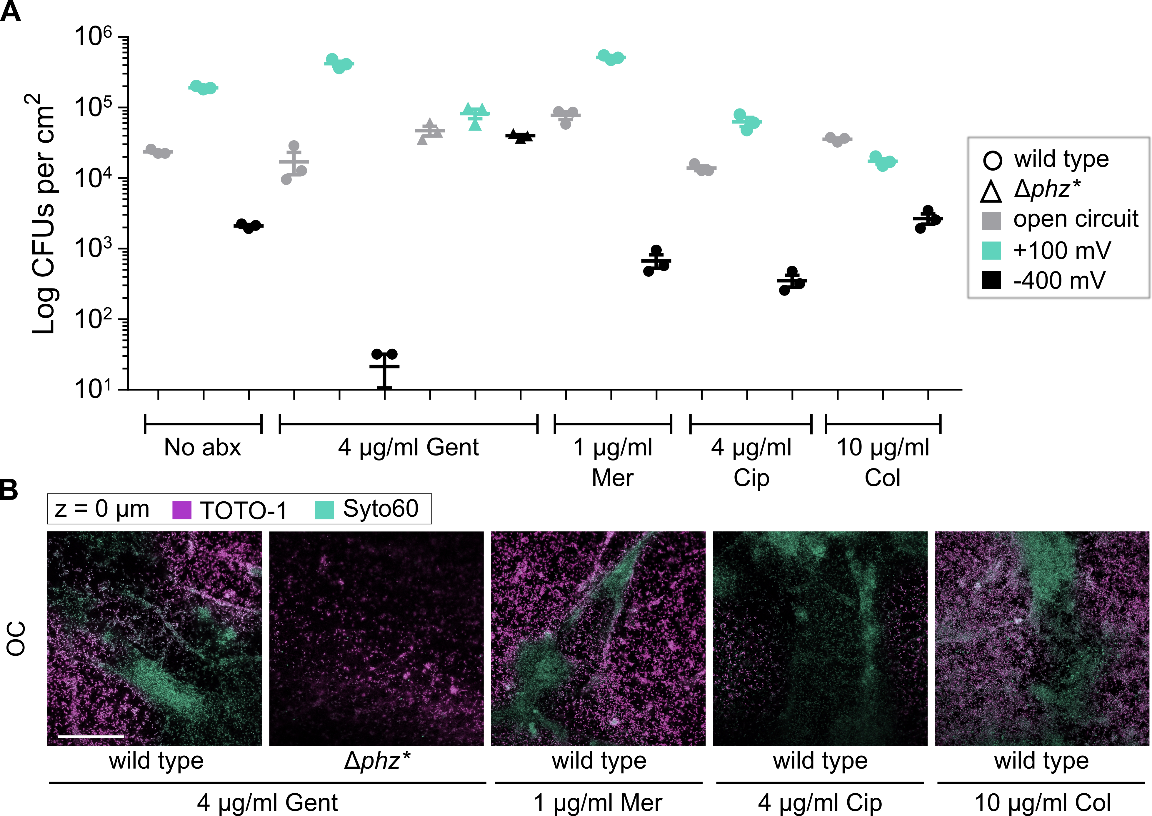


**Figure S4**. Un-normalized data from Fig. 2. **A**) CFUs after 72 hours under anoxic conditions in the presence of either 4 µg/ml gentamicin (Gent), 1 µg/ml meropenem (Mer), 4 µg/ml ciprofloxacin (Cir), or 10 µg/ml colistin (Col), n=3. Error bars represent standard error. Data from samples not treated with antibiotics (No abx) from Fig. 1 plotted again for ease of comparison. **B**) Fluorescence microscopy images of OC samples at biofilm interface with electrode surface using TOTO-1 (cell-impermeable, eDNA) and Syto60 (cell-permeable, all DNA) from representative samples shown in A). Bar = 50 um.
