# Supplementary material for: Pyocyanin-dependent electrochemical inhibition of Pseudomonas aeruginosa biofilms is synergistic with antibiotic treatment
Source: mBio. 2023 Jun 14;14(4):e00702-23. doi: 10.1128/mbio.00702-23 (PMC10470778; doi:10.1128/mbio.00702-23)
Supplement: Fig. S5 — Cell survival over time. [file mbio.00702-23-s0005.docx]

**Supplemental Figure S5**

**
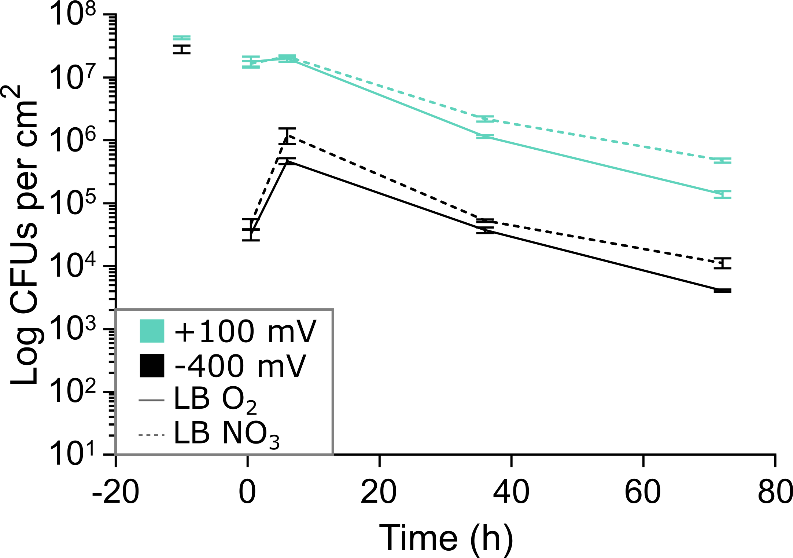
**

**Figure S5**. Cell survival as a funtion of time. t=0 corresponds to transfer to anaerobic reactors and plotted at t=-10 are CFU counts for week-old aerobic biofilms prior to transfer. CFUs/cm^2^ shown for parallel LB O_2_ (line), which were incubated aerobically, and LB NO_3_ (dashed), which were incubated inside an anerobic chamber. CFUs from aerobic biofilms were collected and plated aerobically. Data shown from n=3.
