# Supplementary material for: Pyocyanin-dependent electrochemical inhibition of Pseudomonas aeruginosa biofilms is synergistic with antibiotic treatment
Source: mBio. 2023 Jun 14;14(4):e00702-23. doi: 10.1128/mbio.00702-23 (PMC10470778; doi:10.1128/mbio.00702-23)
Supplement: Fig. S6 — Planktonic culture PYO toxicity. [file mbio.00702-23-s0006.docx]

**Supplemental Figure S6**

**Figure S6**. Toxicity of reduced PYO to liquid-grown cells. Exposure of mid-log aerobic *P. aeruginosa* PA14 liquid cultures to 1, 10, and 100 µM reduced PYO under anoxic conditions. Triplicate data shown normalized to untreated samples with line at mean.
