# Supplementary material for: Pyocyanin-dependent electrochemical inhibition of Pseudomonas aeruginosa biofilms is synergistic with antibiotic treatment
Source: mBio. 2023 Jun 14;14(4):e00702-23. doi: 10.1128/mbio.00702-23 (PMC10470778; doi:10.1128/mbio.00702-23)
Supplement: TABLE S1 — Strains used. [file mbio.00702-23-s0007.docx]

**Table S1. Strains used in this study**

| **Bacterial strain** | **Description** | **Source** |
| --- | --- | --- |
| *P. aeruginosa* UCBPP-PA14 | DKN_WT | Schroth *et al*., 2018 |
| *P. aeruginosa* UCBPP-PA14; Δ*phz** | Δ*phzA1-G1* Δ*phzA2-G2*  Δ*phzMS* Δ*phzH* (derivative of DKN_WT) | Saunders *et al*., 2020 |
