# Supplementary material for: Pyocyanin-dependent electrochemical inhibition of Pseudomonas aeruginosa biofilms is synergistic with antibiotic treatment
Source: mBio. 2023 Jun 14;14(4):e00702-23. doi: 10.1128/mbio.00702-23 (PMC10470778; doi:10.1128/mbio.00702-23)
Supplement: Supplemental Material — Detailed experimental procedures. [file mbio.00702-23-s0008.docx]

**Supplemental Material: Detailed Experimental Procedures**

**Bacterial growth.** *P. aeruginosa* UCBPP-PA14 strains were plated on lysogeny broth (LB) agar from -80°C stocks and incubated at 37°C overnight. Plates were stored for a week at -4°C. Liquid cultures were grown on minimal medium (MM) containing succinate as carbon source and electron donor (14.15mM KH_2_PO_4_, 38.85mM K_2_HPO_4_, 42.8mM NaCl, 9.3mM NH_4_Cl, 40mM Na-succinate, 1x SL-10 trace element solution (1), adjusted to pH 7.2 and autoclaved, then 1mM MgSO_4_ was added) and were incubated while shaking at 37°C.

**Electrochemical reactors and biofilm growth.** Double-jacketed electrochemical reactors (Pine Instruments, 250 ml, wide center port 29/26) were used with ITO-coated 0.2 mm thick boro-aluminosilicate glass slides (Delta Technologies, 24 x 60 mm, Rs = 7-10 Ω) as working electrodes, freshly sanded graphite rods (Alfa Aesar) as counter electrodes, and Ag/AgCl in 3M KCl reference electrodes (BASi). Silver epoxy (Electron Microscopy Sciences) was used to make electrical connection between insulated wire (Digi-key) and working electrode and was insulated using 5 Minute® expoxy (Devcon). Working electrodes were assembled within 5 hours of use. Reactors were sterilized by autoclaving without reference or working electrodes, which were sterilized by submerging in 10% bleach for 5 minutes or 30 seconds, respectively, and rinsed in sterile water. Individual mid-log liquid cultures (OD_600_ 0.45-0.55) were used to inoculate sterile reactors containing 140 ml of MM (described above) with 0.1% (vol/vol) for each biological replicate. Antibiotics were not added at this point. Working electrodes were poised at either open circuit, +100 mV, or -400 mV vs. Ag/AgCl (+310 mV and -190 mV vs the standard hydrogen electrode, respectively). Reactors were aerated using an aquarium pump with a 0.2 µm filter (Thermo Scientific) and heated to 31°C using a circulating water bath (Haake A 25, Thermo Scientific). Fresh medium was exchanged every 24 hours for 5 days. After 5 days of aerobic growth, working electrode-attached biofilms were rinsed in sterile medium to remove planktonic cells and transferred to sterile reactors with fresh MM previously flushed for 2 hours with N_2_ ultra-high purity gas (NRL supply store). For antibiotic exposure experiments, antibiotics were added to sterile media before flushing with N_2_. For PYO-addition experiments, 10 µM PYO (Cayman Chemical) was added to MM before flushing with N_2_. Working electrodes were poised to their corresponding potential and biofilms were incubated for an additional 72 hours under N_2_ flushing at 31°C.

**Biofilm harvesting and processing.** After 72 hours of incubation under anoxic conditions, electrode-attached biofilms were rinsed in sterile media. The biofilms within the bottom 3.12 cm^2^ of each working electrode were scraped off using a cell scraper (Sarstedt) and resuspended in antibiotic-free MM. Serial dilutions were prepared in sterile antibiotic-free MM and plated on antibiotic-free LB. The rest of the electrode-attached biofilms were incubated at room temperature in freshly-made 2 µM TOTO-1 and 10 µM SYTO-60 (Invitrogen) in MM for 15 minutes and rinsed 2x in sterile MM for 15 minutes. Imaging was done with an upright Zeiss LSM 800 microscope with Airyscan and a 40x oil immersion lens. SYTO-60 was excited with a 561 nm; 0.20% laser and emission was recorded from 580-700 nm. TOTO-1 was excited with a 488 nm; 0.20% laser and emission was recorded from 490 to 580 nm.

**Square Wave Voltammograms.** Potential was scanned from +100 mV to -500 mV vs Ag/AgCl and in reverse with the following paramenters: pulse height = 50 mV, pulse width = 66.6 ms, step height =-0.5 mV, and acquisition of the average current over the last 50% of each step. Square wave voltammograms were acquired immediately after transfer from oxic to anoxic conditions.

**Biofilms on unpoised electrodes within oxidative/reductive reactors.** Electrochemical reactors were prepared as described above, except that an additional ITO-covered glass slide identical to the working electrode was insterted throught the central port, but it was never connected to the potentiostat. The wires of the working electrode and the unpoised slide were positioned so that the distance between them was ~3cm. Biofilms grown on unpoised slides were therefore subjected to the same diffusive conditions as the biofilms grown on poised working electrodes, but without direct contact with it. Both poised ans unpoised electrodes were transferred together to anoxic reactors after 5 days of growth and processed at the same time after harvesting.

**Biofilms harvested and plated in parallel oxic/anoxic media.** For experiments analyzing cell survival as a function of time, aerobic biofilm growth and transfer to anoxic reactors was performed as described above. Time point t=0 corresponded to when working electrodes were re-poised to their corresponding potentials after transfer to anoxic conditions. At t= 30 minutes, 6, 36, and 72 hours, anoxic electrochemical reactors were transferred into an anaerobic chamber (Whitley Workstation DG250) with a 7%:20%:73% H_2_:CO­_2_:N_2_ atmosphere where biofilm harvesting was performed as described above, except that the MM used to resuspend biofilms and prepare serial dilutions contained 2 g/L bicarbonate and was flushed with a 80%:20% N_2_:CO_2_ gas mix (Airgas) prior to sterilization and equilibrated inside the chamber for at least 24 hours. Anoxic LB plates were prepared with 40 mM KNO_3_ as electron acceptor, pH was adjusted to 7.0, then 2 g/L bicarbonate was added before flushing with 80%:20% N_2_:CO_2_ and autoclaving. Plates were poured inside the anaerobic chamber and equilibrated for 24 hours before use. Oxic LB plates were brought into the anaerobic chamber for parallel plating, but were transferred back out immediately after plating and were incubated as described above.

**Treatment of liquid cultures with reduced PYO.** 100 µM PYO in MM was reduced electrochemically using a dual-chamber reactor with an cation exchange membrane (CMI-7000, Membranes International) to avoid re-oxidation of PYO at the counter electrode. Anodic chamber contained Ag/AgCl reference electrode, platinum mesh working electrode, and was flushed with N­_2_; while cathodic chamber was oxic and contained platinum mesh counter electrode. PYO reduction was carried out by poising the working electrode at -400 mV vs. Ag/AgCl for 24 hours, past the point of 100% PYO reduction based on coulombic efficiency, and biochemical reactor was then transferred into the anaerobic chamber. Anoxic vials with pre-equilibrated MM and corresponding concentrations of reduced PYO were then treated with biochemical O_2_ scavenging system (10mM glucose + 375nM glucose oxidase + 750nM catalase) for an hour before inoculation. Aerobic liquid cultures of *P. aeruginosa* in MM at mid-log (OD600 = 0.5) were spun down and resuspended in fresh media and used to inoculate assays with 10% (vol/vol). After 30 minutes, serial dilutions were prepared using anoxic MM and plated for CFU counts in LB + KNO_3_ as described above.

**Supplemental References**

1. Atlas RM. 2004. Handbook of microbiological media.3rd Edition. CRC Press, Boca Raton, FL.
